# Involvement of hyaluronidases in colorectal cancer

**DOI:** 10.1186/1471-2407-10-499

**Published:** 2010-09-17

**Authors:** Helen Bouga, Isidoros Tsouros, Dimitrios Bounias, Dora Kyriakopoulou, Michael S Stavropoulos, Nikoletta Papageorgakopoulou, Dimitrios A Theocharis, Demitrios H Vynios

**Affiliations:** 1 Laboratory of Biochemistry, Department of Chemistry, University of Patras, Patras, Greece; 2 Department of Surgery, University Hospital, Patras, Greece; 3 Laboratory of Biological Chemistry, Department of Medicine, University of Patras, Patras, Greece

## Abstract

**Background:**

Hyaluronidases belong to a class of enzymes that degrade, predominantly, hyaluronan. These enzymes are known to be involved in physiological and pathological processes, such as tumor growth, infiltration and angiogenesis, but their exact role in tumor promotion or suppression is not clear yet. Advanced colorectal cancer is associated with elevated amounts of hyaluronan of varying size. The aim of the present study was therefore to illuminate the importance of hyaluronidases in colon carcinoma progression.

**Methods:**

The patients' samples (macroscopically normal and cancerous) were subjected to sequential extraction with PBS, 4 M GdnHCl and 4 M GdnHCl - 1% Triton X-100. The presence of the various hyaluronidases in the extracts was examined by zymography and western blotting. Their expression was also examined by RT-PCR.

**Results:**

Among hyaluronidases examined, Hyal-1, -2, -3 and PH-20 were detected. Their activity was higher in cancerous samples. Hyal-1 and Hyal-2 were overexpressed in cancerous samples, especially in advanced stages of cancer. Both isoforms were mainly extracted with PBS. Hyal-3 was observed only in the third extract of advanced stages of cancer. PH-20 was abundant in all three extracts of all stages of cancer. The expression of only Hyal-1 and PH-20 was verified by RT-PCR.

**Conclusion:**

A high association of hyaluronidases in colorectal cancer was observed. Each hyaluronidase presented different tissue distribution, which indicated the implication of certain isoforms in certain cancer stages. The results provided new evidence on the mechanisms involved in the progression of colorectal cancer.

## Background

Hyaluronan (HA) is a multifunctional high molecular mass (HMM) glycosaminoglycan responsible for the maintenance of the extracellular matrix (ECM) of connective tissue. It is a simple linear glycosaminoglycan (GAG) composed of repeating disaccharide units of D-glucuronic acid and N-acetyl-D-glucosamine: [-*β*(1,4)-GlcUA-*β*(1,3)-GlcNAc-]_n_. It is normally produced by hyaluronan synthases (HAS1, HAS2, HAS3) at plasma membrane and degraded extracellularly by the action of plasma membrane hyaluronidases (Hyals) [1-3], endocytosed, transferred to lysosomes where it is fully degraded by the action of hyaluronidase, beta-glucuronidase and beta-N-acetylglucosaminidase. Depending on the tissue source, HA usually consists of 2,000-25,000 disaccharide units, giving rise to molecular mass ranging from 10^6 ^to 10^7 ^Da [4,5]. Its lower molecular mass (LMM) forms participate in a wide variety of biological functions. HMM-HA is indicative of healthy tissues, while LMM-HA seems to promote angiogenesis and activate signaling pathways that are critical for cancer progression. The LMM fragments could be truncated products of the synthetic reaction, but could also be the result of hyaluronidase activity [6].

Due to its unique biophysical properties, HA contributes directly to tissue homeostasis, interacts with link proteins and proteoglycans (PGs) thus maintaining the structural integrity of extracellular and pericellular matrices, and its interaction with cell surface HA receptors mediates crucial influences of HA on cell behavior. Due to all of these functions, HA plays regulatory roles in basic cellular behavior, such as cell adhesion, cell migration, cell-cell recognition and cell differentiation [7,8], and thus participates in many important processes of morphogenesis, tissue remodeling, inflammation and several types of diseases, such as tumor growth and atherosclerosis. Elevated extracellular amounts of HA and its partially catabolized oligomers are correlated with several types of malignancies potentially due to decoupled synthesis and degradation [9,10].

Hyaluronidases (Hyals) are a class of enzymes that degrade, predominantly, HA, and at a slower rate chondroitin and chondroitin sulphate. Hyals are endoglycosidases that degrade the β-N-acetyl-D-glucosaminidic linkages in HA chains [11]. In the human genome there are six known genes coding for hyaluronidase-like sequences, all having a high degree of homology, but with different tissue distribution, namely hHyal-1 through -4, PH-20/Spam-1 and pseudogene Phyal1, that is transcribed in the human genome but not translated. Human Hyal-1 and Hyal-2 are the two major Hyals for the degradation of HA in somatic tissues, hHyal-2 degrades high molecular mass HA to an approximately 20 kDa product, whereas Hyal-1 can degrade high molecular mass HA to small oligomers, primarily to tetrasaccharides [12]. A product of the human *hyal-4 *gene, Hyal-4, based on preliminary studies, is also a chondroitinase with a predominant activity toward Ch and ChS. According to their pH activity profiles, they are divided in two categories; Hyal-1, Hyal-2 and Hyal-3 are active at acidic pH (pH 3.0-4.0) and are considered as acidic Hyals [13], while PH-20 is a neutral active Hyal, as it is active at pH 5.0-8.0 [14].

Hyals are known to be involved in biological processes such as development and tumorigenesis [13,15]. Among the six mammalian Hyals, Hyal-1 is the major tumor-derived Hyal and is expressed by a variety of tumor cells, confirmed with several methods (RT-PCR analysis, cDNA cloning, protein purification, immunoblotting and immunohistochemistry). In addition to Hyal-1, RT-PCR analysis has revealed PH-20 expression in head and neck carcinoma, especially laryngeal carcinoma [16,17]. Hyals levels are also shown to be elevated in breast tumors and RT-PCR analysis has detected the expression of PH-20, Hyal-2 and Hyal-3 in breast cancer tissues [18,19]. These observations suggest that Hyals appear to be implicated in many carcinomas, however the exact role of Hyals in colon cancer remains under great concern.

Accumulating evidence has demonstrated that the production of HA is excessive in malignant cancers; increased HA serum levels and deposition in tumour tissue are often associated with malignant progression in colorectal cancer [20]. The purpose of this study was to examine the activity and expression of Hyals in tissue samples from patients with colorectal cancer and to identify different isoforms profile in different stages of cancer.

## Methods

### Chemicals

Phosphate buffer saline (PBS), phenylmethylsulfonyl fluoride (PMSF), Alcian blue and Coomassie Brilliant blue were obtained from Serva (Darmstadt, Germany). Hyaluronic acid, hyaluronidase (type I) from bovine testes, benzamidine hydrochloride, ε-amino-n-caproic acid, Triton X-100, N-ethylmaleimide (NEM) and ethylenediaminetetracetic acid disodium salt dihydrate (Na_2_EDTA) were obtained from Sigma. Mouse polyclonal antibodies against hyaluroglucosaminidase 1 (Hyal-1), hyaluroglucosaminidase 3 (Hyal-3) and sperm adhesion molecule 1 (PH-20/Spam-1) were obtained from Abnova Corporation (Taiwan). RNA extraction kit (Nucleospin RNA II) was from Macherey-Nagel. PrimeScript™one step RT-PCR kit and 100 bp DNA ladder were obtained from TakaRa BIO INC. All other chemicals used throughout the study were of the highest available grade.

### Tissue source

Macroscopically normal and cancerous tissues were obtained from 34 patients (8 females and 26 males, age range 45-90), who underwent surgical operation due to colorectal carcinoma at the Surgical Clinic of the General University Hospital of Patras, Greece. The patients included in the study were healthy until the time of diagnosis of colorectal cancer, without any other disease. Surgical procedures consisted of different types of colorectal resection. Two specimens were obtained from each patient, one from the center of the tumor and the other of similar weight from areas adjacent to the cancerous regions (macroscopically normal areas), and were stored at -80°C for further biochemical examination. Clinical information was obtained after clinical and pathological diagnosis of the patients: sex, age, location of primary tumor, pathological differentiation, distant metastasis and radiation therapy (Table 1). The clinical stage of all tumors was completed according to TNM and Astler-Coller (A/C) classification. The study design was approved by the Ethical Committee of the University Hospital of the University of Patras and informed consent was obtained from all patients before entry into the study.

**Table 1:** Characteristics of patients with colon cancer.

Case no.	Age	Sex	**Loc**^**1**^	**Hist. features**^**2**^	**A/C stage**^**3**^
1	59	M	S	-	Tis
2	66	M	C	-	Tis
3	61	M	R	-	Tis
4	72	F	T	Well	A
5	90	M	R	Poor	B1
6	76	M	S	Med	B1
7	56	M	R	Med	B1
8	54	F	S	Well	B1
9	74	F	S	Med	B2
10	73	M	D	Med	B2
11	84	F	D	Med	B2
12	81	M	R	Poor	B2
13	66	M	C	Med	B2
14	74	M	S	Well	B2
15	77	M	S	Med	B2
16	79	M	C	Med	B2
17	45	M	T	Med	B2
18	58	M	S	Med	B2
19	60	F	R	Med	C1
20	82	F	R	Med	C1
21	65	M	C	Med	C1
22	59	M	R	Med	C1
23	68	F	R	Med	C1
24	78	M	S	Med	C2
25	87	M	R	Med	C2
26	71	M	R	Med	C2
27	72	M	A	Poor	C2
28	57	M	S	Med	C2
29	61	M	A	Med	C2
30	50	M	R	Med	C2
31	80	M	C	Med	C2
32	80	F	A	Poor	C2
33	72	M	S	Med	D
34	75	M	A	Poor	D

*The patients have been classified according to the stage of cancer*.

^1^Location (of primary tumor); A: ascending colon; C: cecum; D: descending colon; R: rectum; S: sigmoid colon; T: transverse colon.

^2^Histological features (Grade of differentiation); well: well differentiated; mod: moderately well differentiated; poor: poorly differentiated.

^3^A/C stage; Astler-Coller classification of tumors. - represents non cancerous samples (adenoma tissues).

### Extraction and Zymographic examination of Hyals

Parts of macroscopically normal and cancerous tissues were used for the detection of Hyals. Each specimen was finely diced and the macromolecules contained were sequentially extracted for 3 × 24 h periods at 4°C in the dark with PBS (10 mM disodium phosphate, 0.14 M NaCl, pH 7.4), 4 M GdnHCl - 0.05 M sodium acetate and 4 M GdnHCl - 0.05 M sodium acetate - 1% Triton X-100, using 10 vols of extraction buffer per g of tissue [21]. This three-step extraction procedure was applied to permit the differential extraction of Hyals isoforms, since accumulated evidence suggests that they are present in either soluble or membrane-bound form [11,12]. A protease inhibitor cocktail was included containing 5 mM benzamidine hydrochloride, 0.4 mM phenylmethylsulfonyl fluoride, 10 mM N-ethylmaleimide, 0.1 M ε-amino-n-caproic acid and 0.01 M Na_2_EDTA. Each one of the extracts was stored at -20°C until use.

Hyaluronidase activity of the various extracts was examined using a HA zymography procedure, as described previously [22-24]. The samples were electrophoresed and at the end of the electrophoresis the gel was submerged in the suitable buffer (0.15 M NaCl-0.1 M CH_3_COONa pH 3.7) and incubated at 37°C for 16 h. Then the gel was stained sequentially with Alcian blue to stain undegraded HA and with Coomassie blue to overstain the Alcian blue stained HA and the non enzymic protein bands. The enzyme activity appeared as white bands in a dark blue background. Finally, semi-quantification of the enzymatic units was achieved after scanning of the gel by a digital scanner.

### Western Blotting identification of Hyaluronidases

The various extracts were subjected to SDS-PAGE (T: 10%, C: 2.7%) according to Laemmli [25], using denaturing conditions in the presence of β-mercaptoethanol. After electrophoresis, the gels were immersed in 0.15 M iodoacetamide in 0.05 M Tris-HCl pH 8.3 for 15 min at room temperature to block free β-mercaptoethanol. Thereafter, the protein bands were electrotransferred to nitrocellulose (Immobilon NC) membranes at constant current of 80 mA at 4°C for 20 h in 0.05 M Tris-HCl pH 8.3. The membranes were washed with PBS containing 0.1% Tween 20 (PBS-T) and blocked with 5% dry skimmed milk in PBS-T. They were then incubated with the respective polyclonal antibody (against either Hyal-1, Hyal-2, Hyal-3 or PH-20) in an appropriate dilution in PBS-T for 1 h at room temperature. After repeated washings with PBS-T, the membranes were incubated with second antibody (goat anti-mouse IgG) peroxidase-conjugated (1:5000) in PBS-T, for 1 h at room temperature and washed exhaustively with PBS-T. The immunoreacting bands were visualized by enhanced chemiluminescence method (ECL), according to the manufacturer's instructions and by exposure to Agfa Curix X-ray film.

### RNA extraction and RT-PCR analysis

Colorectal specimens were pulverized in liquid nitrogen and subjected to total RNA extraction, using the Nucleospin extraction kit, as described by the manufacturer and treated with RNase-freeDNase to remove contaminating genomic DNA. Strand cDNA was synthesized from 40 ng of total RNA in 50 μl reaction components for one-step RT-PCR kit, according to the manufacturer's instructions. This reaction mixture contained in addition 1 μM of the sense and antisense primers showed in Table 2. The amplification was performed in a GeneAmp 2400 thermal cycler (Perkin-Elmer Co.) and the reaction profile used for all primers sets was: 95°C for 10 min for the activation of DNA polymerase and then 25-35 cycles, depending on the analysis, at 94°C for 30 s, 62°C for 1 min, and 72°C for 10 min to finalize extension. The number of cycles was chosen so that reactions could be terminated during the linear phase of amplification. The reaction products were separated by electrophoresis in 2% (w/v) agarose gels contained Gelstar Stain to visualize the amplified cDNA fragments under UV. The gels were then scanned and the bands were analyzed densitometrically. Quantitative differences between cDNA samples were normalized by including GAPDH in all experiments.

**Table 2:** Sequences of oligonucleotide primers for RT-PCR analysis.

Primer	Sequence (5'-3' direction)	Product length (bp)
Hyal-1 (forward)	CATATTGAGAACCTAATGCACTCTG	208
Hyal-1 (reverse)	GGAATGAATGGTGTCTGCTGTGG	
Hyal-2 (forward)	TTGTGAGCTTCCGTGTTCAG	217
Hyal-2 (reverse)	GTCTCCGTGCTTGTGGTGTA	
PH-20 (forward)	GAGTTGTAAGGAGAAAGCTGAT	194
PH-20 (reverse)	TGGCTACAGAAGAAATGATAAGAAACA	
GAPDH (forward)	TCAACGGATTTGGTCGTATTGGG	270
GAPDH (reverse)	GACTCCACGACGTACTCAGC	

### Statistical analysis

Statistically significant differences in the detection of Hyals between healthy, macroscopically normal and cancerous tissues, were executed by students' *t*-test, using the microcal origin software. Significance was determined at values of P < 0.05.

## Results

### Hyaluronidase activity of tissue extracts in colon cancer

**Figure 1:**
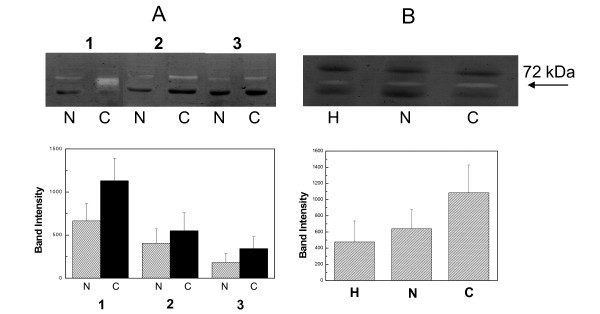
**Hyaluronidase extractability**. A. Up. Typical hyaluronan zymography pattern of the three sequential extracts of the samples, macroscopically normal (N) and cancerous (C) of patients with colorectal cancer. The extracts are PBS (1), 4 M GdnHCl (2) and 4 M GdnHCl - 1% Triton X-100 (3). Down. Semi-quantitative representation of the zymography experiments. B. Up. Hyaluronidase activity in PBS extracts from healthy subjects (H), macroscopically normal (N) and cancerous (C) specimens of patients. Down. Semi-quantitative representation of the zymography experiments. The arrow indicates the migration of the main lysis band.

The sequential extracts from the various specimens were subjected to hyaluronan zymography in acidic conditions. In the majority of samples one lysis band was observed corresponding to 72 kDa. In minor number of samples, bands of smaller molecular mass (20-40 kDa) were also identified (not shown). These samples were of advanced stages of cancer (stages B and C). Semi-quantitative analysis of the zymograms revealed increased hyaluronidase activity in cancerous PBS tissue extracts compared to the macroscopically normal PBS extracts (fig. 1A), in all anatomic sites examined (not shown). Both activities were significantly increased compared to intact, healthy tissues (fig. 1B).

**Figure 2:**
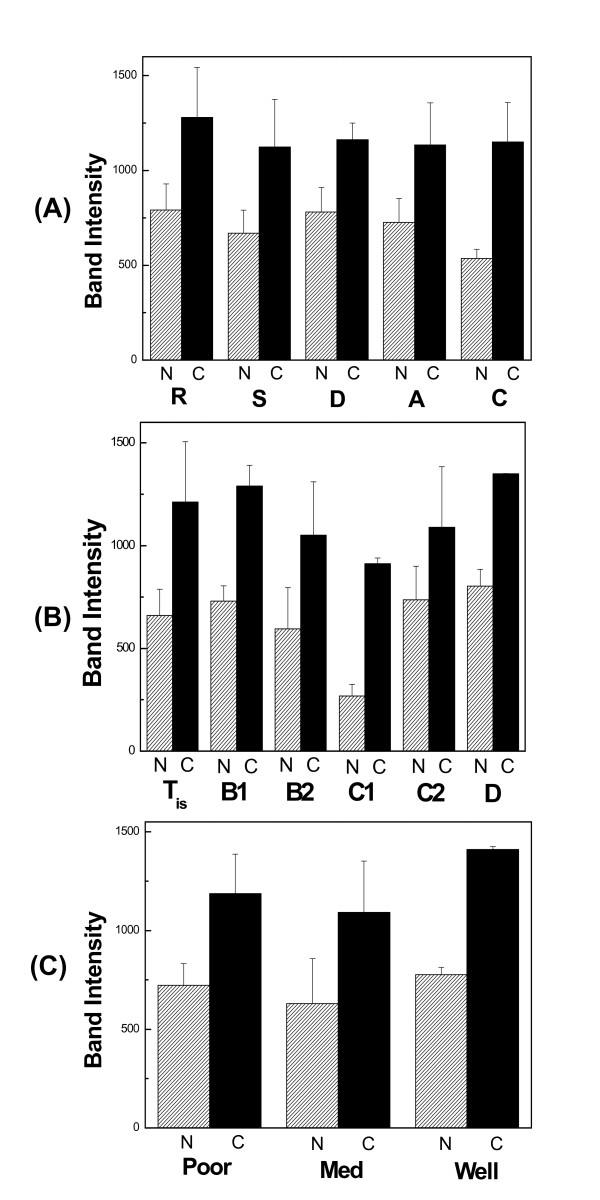
**Quantification of hyaluronidase activity**. Semi-quantitative analysis of hyaluronidase in colon cancer after zymography of PBS extracts according to: (A) anatomic site, (B) stage of cancer, and (C) grade of cancer.

Hyaluronidase activity was significantly elevated in cancerous specimens compared to macroscopically normal ones in all anatomic sites examined (fig. 2A). Especially in the case of sigmoid and cecum, an increase of about 80 and 100% was calculated, whereas the increase was much smaller in the other anatomic sites. In addition, hyaluronidase activity seemed to vary with the stage of cancer. The highest activity was observed at early stages (Tis/A), as well as at stage D of cancer and the lowest activity at. stage C1 (fig. 2B). Hyaluronidase activity was also related to the grade of cancer, being obvious in cancerous samples (fig. 2C), whereas the macroscopically normal samples seemed to contain about the same activity of the enzyme.

**Figure 3:**
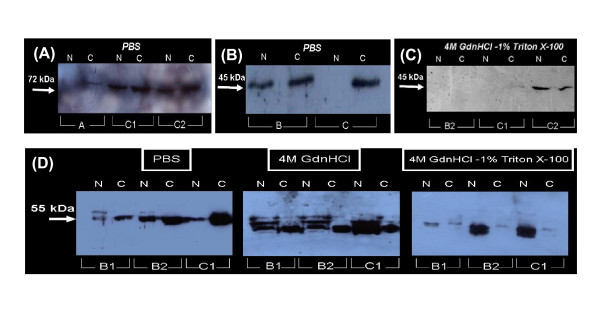
**Western blotting analysis of hyaluronidases**. A. Hyal-1 in macroscopically normal (N) and cancerous (C) PBS extracts of A, C1 and C2 stages of cancer. B. Hyal-2 in macroscopically normal (N) and cancerous (C) PBS extracts of B and C stages of cancer. C. Hyal-3 in macroscopically normal (N) and cancerous (C) 4 M GdnHCl-1% Triton X-100 extracts of B2, C1 and C2 stages of cancer. D. PH-20 in macroscopically normal (N) and cancerous (C) samples of B2, C1 and C2 stages of cancer; left; PBS extracts, middle; 4 M GdnHCl extracts and right; 4 M GdnHCl-1% Triton X-100 extracts. The arrows indicate the migration of the main immunoreacting band.

Western blotting analysis was further used for the identification of Hyal(s) present in the sequential extracts. In all tissue samples examined, Hyal-1, Hyal-2, Hyal-3 and PH-20 were found to exist, each one observed in different extracts and different stages of cancer. Hyal-1 was overexpressed in cancerous samples than in macroscopically normal ones, mainly in advanced stages of cancer (fig. 3A). Hyal-2 was expressed, but not in large amounts, in tissue samples of patients with advanced stage (B and C) of cancer (fig. 3B). Both Hyal-1 and Hyal-2 were mainly, if not exclusively, extracted with PBS. On the other hand, Hyal-3 was observed only in 4 M GdnHCl-1% Triton X-100 extracts of advanced stages of cancer (fig. 3C). PH-20 was abundant in all three extracts of different stages of cancer; in PBS extracts PH-20 was detected mainly in cancerous samples (fig. 3D), but in 4 M GdnHCl and 4 M GdnHCl-1% Triton X-100 extracts was mainly detected in macroscopically normal samples.

### RT-PCR analysis of hyaluronidase transcripts in colon cancers

**Figure 4:**
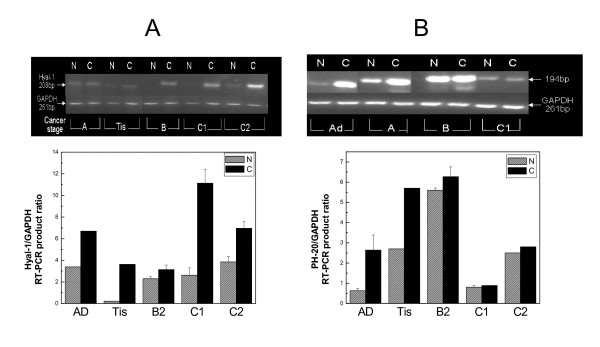
**RT-PCR analysis of hyaluronidases**. A. Up. cDNA product of Hyal-1 (208 bp) in macroscopically normal (N) and cancerous (C) samples from different patients. Down. Semi-quantitative representation of the results. B. Up. cDNA product of PH-20 (194 bp) in macroscopically normal (N) and cancerous (C) samples from different patients. Down. Semi-quantitative representation of the results. The arrows indicate the migration of cDNA products of GAPDH and hyaluronidases.

Hyaluronidases expression in tissue was also examined by RT-PCR analysis (fig. 4). The results indicated that Hyal-1 was overexpressed in cancerous samples compared to macroscopically normal ones. Semi-quantitative analysis of the results revealed increased Hyal-1 expression especially in B2, C1 and C2 stage of cancer (fig. 4A). PH-20 was overexpressed at early stages of cancer, especially in the cancerous samples; considerable expression was also observed in benign tumors (fig. 4B). Hyal-2 and -3 expression at the mRNA levels was not identified at any of the samples examined. Additional examinations, regarding any mutations of the genes or alternative splicing of mRNA have not been performed.

## Discussion

A large amount of studies shows that hyaluronan deposition is up-regulated in most malignancies. Especially in human cancers, hyaluronan concentrations are usually higher in cancerous than in normal tissues. Colon cancer, among other kinds of cancer, is considered to enriched with hyaluronan [6,26]. In that kind of tumor, hyaluronan may support tumor growth by stimulating anchorage-independent growth and proliferation of tumor cells. Moreover, hyaluronan may also actively promote tumor cell adhesion, migration and metastasis and may also protect against immune surveillance [27]. Additionally, tumor cells may take advantage of hyaluronan-rich extracellular matrices to invade more easily into the surrounding tissues.

Based on these observations, in this work we investigated the activity and the expression of hyaluronidases in tissue samples from patients with colon cancer, for clarifying the dependence of hyaluronan catabolism on individual hyaluronidase activities under pathological conditions. It has been reported that hyaluronidases act as tumor inhibitors in vivo [28], Hyal-1 reversibly correlates with hyaluronan content [29] and that Hyal-1 correlates with tumor grade, stage, and multifocality [17,30]. The results of our study indicated that hyaluronidases were involved in colon cancer progression. Sequential extraction of hyaluronidases from the various specimens was applied in the study to obtain an idea on their localization, since PBS would extract enzyme isoforms freely available within the tissue, 4 M GdnHCl would extract enzyme isoforms entrapped in or interacted with macromolecular aggregates and Triton-X would extract membrane bound or intracellularly localized enzyme isoforms.

At first, much more activity of hyaluronidase was observed in PBS extracts, compared to 4 M GdnHCl and 4 M GdnHCl-1% Triton X-100 extracts. About 50% of the enzyme activity was extracted with PBS from the macroscopically normal specimens, and more than 60% from the cancerous. This observation suggested that hyaluronidases were not tightly bound to the tissue or the cells. By comparing the total hyaluronidase activity in healthy colon, and in macroscopically normal and cancerous specimens of colorectal carcinoma samples, it was found to be elevated up to 100% in cancerous specimens compared to both others.

Statistical analysis of the results obtained from hyaluronan zymography revealed that hyaluronidase activity was related to the stage and grade of cancer. Specifically, at early stages high hyaluronidase activity was observed, and after stage B2 the enzymatic activity declined, however it increased at very late stages. These results implied that hyaluronidases in the onset of pathological conditions tended to create small fragments of hyaluronan that help tumor progression. It would be interesting to identify the size distribution of hyaluronan at early stages of cancer, since it is well reported that hyaluronan of small molecular mass is present in cancer [31,32].

On the other hand, high molecular mass hyaluronan induces tissue hydration and therefore it physically creates spaces through which tumor cells migrate and invade, and this might be the explanation of the decreased hyaluronidase activity after the early stages. The increase of the activity at late stages of cancer may be required for the final degradation and the removal of the ECM components sensitive to the enzyme. Of course, it is not only hyaluronidases activity, but hyaluronan synthases activity that plays also important role in hyaluronan size and therefore in tumor progression and this is another point of inerest. A third point requiring clarification is concerning to the presence of highly expressing CD44 cells, being an additional significant factor related to hyaluronan that affects tumor progression.

As far as it concerns the grade of cancer it seemed that there was no significant differences neither between macroscopically normal samples of poor, moderately and well differentiated specimens nor between cancerous ones. Nevertheless, cancerous specimens contained 50-80% more hyaluronidase activity than macroscopically normal.

Using Western Blotting analysis, we managed to clear up the involvement of individual hyaluronidases existing in certain parts of the tissue; normal and cancerous. The hyaluronidases existing in samples were Hyal-1, -2, -3 and PH-20. It seemed that at early stages of tumor growth PH-20 was the most abundant extracellularly, as it was extracted mainly with PBS. At advanced stages it seemed that Hyal-1, -2 and PH-20 had a collaborating action for fragmention of hyaluronan. The above hyaluronidases didn't act just extracellularly, but also had a transmembrane action, since they appeared in PBS and 4 M GdnHCl extracts. The late stages of cancer (C2 and D) seemed to prefer mostly the synergistic action of Hyal-1, PH-20 and Hyal-3. The last one seemed to be localized entirely in cell membrane as it was present only in 4 M GdnHCl-1% Triton X-100 extracts. However, it remained unclear at what point the fragmentation of hyaluronan switched from an extracellular or cell surface process to an endosomal or lysosomal process and might be due to the energy demands of the cancer cells. In addition, the factors regulating the differential expression of hyaluronidase isoforms are not known, although it has been proposed growth factors may have such effect [33] and Hyal-1 is regulated epigenetically [34].

## Conclusions

The metabolic pathways for hyaluronan degradation are highly ordered, composed of carefully controlled reactions that rely on regulation of individual enzyme activities. From our work hyaluronidases seemed to play different tissue distribution role in colon cancer, with a combining action of these isoenzymes in colon cancer progression. Understanding the mechanisms of hyaluronidases involved in colon cancer progression may be a useful tool for future prognosis of colon cancer. However, how such regulation and mechanisms are accomplished needs to be clarified.

## Competing interests

The authors declare that they have no competing interests.

## Authors' contributions

HB, IT and DHV carried out RT-PCR analysis, western blotting and data analysis, participated in the study design and drafted the manuscript. DB, DK and MSS participated in the collection and characterization of patiens' samples. NP and DAΤ conceived of the study and participated in its design and coordination. All authors read and approved the final manuscript.

## Pre-publication history

The pre-publication history for this paper can be accessed here:

http://www.biomedcentral.com/1471-2407/10/499/prepub
